# Associations of transcription factor 7-Like 2 (*TCF7L2*) gene polymorphism in patients of type 2 diabetes mellitus from Khyber Pakhtunkhwa population of Pakistan

**DOI:** 10.4314/ahs.v21i1.4

**Published:** 2021-03

**Authors:** Taha Hameed, Zahid Khan, Muhammad Imran, Saif Ali, Abdullah Abdo Albegali, Muhammad Ikram Ullah, Hasan Ejaz

**Affiliations:** 1 Department of Biochemistry, University of Peshawar, Pakistan; 2 Department of Pharmacy, The University of Lahore, Pakistan; 3 Department of Clinical Laboratory Sciences, College of Applied Medical Sciences, Jouf University Sakaka, Saudi Arabia

**Keywords:** T2DM, TCF7L2, Genetic association, ARMS-PCR, Single nucleotide Polymorphism (SNPs), Khyber Pakhtunkhwa

## Abstract

**Background:**

Type 2 diabetes mellitus (T2DM) is the most prevalent component of metabolic syndrome. Environmental factors and various complex genes like transcription factor 7-like 2 (*TCF7L2*) gene have involved in the disease development.

**Objective:**

To determine *TCF7L2* genetic association (rs7903146C/T and rs12255372G/T) in T2DM patients of Khyber Pakhtunkhwa population of Pakistan.

**Subjects and methods:**

This study comprised of 176 subjects including 118 T2DM patients and 58 healthy controls. Genomic DNA was extracted and genotype of common variants (rs7903146 C/T and rs12255372 G/T) was carried out by amplification-refractory mutation system (ARMS)-PCR of sequence specific oligonucleotides.

**Results:**

The distribution of genotype of *TCF7L2* SNPs (rs7903146 C/T and rs12255372 G/T) was significantly associated with T2DM as compared to the controls (p <0.0001). The genetic models of the rs7903146 (C/T) and rs12255372 (G/T) SNPs were significantly associated between cases and controls (p <0.0001). On the other hand, the significant association was observed between the two SNPs and different biochemical parameters like serum fasting glucose, lipid profile, creatinine and blood HbA1c levels (p <0.05).

**Conclusion:**

It is concluded that the SNPs of the *TCF7L2* gene are significantly associated with T2DM disease susceptibility in the population of Khyber Pakhtunkhwa of Pakistan.

## Background

Diabetes mellitus (DM) is one of the commonest metabolic disorder which is characterized by having persistent hyperglycemia due to the abnormalities in insulin secretion or resistance to the insulin action^1^. The burden of DM is increasing in the developing countries including South Asian populations. Type 2 diabetes mellitus (T2DM) is the most frequent type of diabetes which can develop metabolic syndrome due to the basic dysfunctions of insulin (resistance or lack of secretion). Insulin resistance in T2DM along with hypertension, obesity and dysipidemia is the major risk factor for metabolic syndrome. In Pakistan, there are conflicting reports for the prevalence of T2Dm which ranges from 7–19%^2^,^3^. In Khyber Pakhtunkhwa region, the prevalence reported for T2DM is about 9%^3^. T2DM is the multifactorial anomaly including numerous environmental, metabolic and complex genetic risk factors. Various studies have been conducted for the susceptibility of T2DM, but very few reports are available from the South Asian populations^4^. South Asian populations are very genetically heterogeneous and comprised of Pakistan, India and Bangladesh countries. In South Asians resident of UK, it has been demonstrated that genetic variants can affect more than 10% of population which is six times more type 2 diabetes than Caucasian population^5^.

Several studies investigated the association of genetic variants including *TCF7L2* that could develop T2DM^6^,^7^. Genetic polymorphisms of the *TCF7L2* have been strongly linked to T2DM susceptibility and more reproducible association with disease than any other reported genes^8^–^10^. *TCF7L2* gene is localized on chromosome 10q25 and it encodes 215.9 kb nucleotide sequence^11^. This gene plays role in Wnt-signaling pathway^12^ and affects the insulin resistance^13^. Although, *TCF7L2* is considered to play function in insulin secretions from pancreas but the exact mechanism for the gene involvement in diabetes development is unclear^6^,^11^,^13^–^14^. Genetic polymorphism of *TCF7L2* gene has been widely investigated in different populations like Chinese, White Europeans, Israeli, African-American, Argentinians, West.

Africans, Mexicans, Indians, Iranians, and Pakistani groups^13^–^18^. On the other hand, some other studies described the lack of association between SNP variants with type 2 diabetes^19^–^20^.

From Pakistan, some previous reports documented the association of *TCF7L2* SNPs with T2DM^21^,^23^ and some other data did not find the link to T2DM^20^. Pakistani population is a complex ethnic group with different language speaking and cultures. Very few studies have been conducted for the role of *TCF7L2* with T2DM in this population. Till present, no published data documented the role of *TCF7L2* SNPs in diabetes risk for Pashtun language group of Pakistan. Therefore, this study was aimed to determine the association of common SNPs (rs7903146 C/T and rs12255372 G/T) of *TCF7L2* with susceptibility of type 2 diabetes in population of Northern region of Pakistan from the Khyber-Pakhtunkhwa province.

## Subjects and methods

Ethical approval of this research was granted from the institutional research board (IRB) of Lady Reading Hospital (LRH) and University of Peshawar, Pakistan (IBR/UoP/2017/7817). Helsinki guidelines (2008) were followed for sample collection of human subjects after written informed consent.

### Subject selection and sample collection

Sample size calculation carried by online tool (The Survey System Creative, Research Systems). The sample size was calculated by the following formula keeping the confidence level equal to 95% and the margin of error equal to 7%. The calculated sample size for each group was 55 subjects. This study comprised of total 176 subjects including 118 T2DM patients and 58 healthy controls. The cases were recruited from Lady reading hospital, Peshawar and healthy controls were obtained from same ethnic region of Peshawar. T2DM cases were selected according to the prescribed criteria by American Diabetes Association (fasting plasma glucose (FPG) ≥ 126 mg/dL, random plasma glucose of 200 mg/dL or impaired oral glucose tolerance test OGTT (2-hour plasma glucose ≥ 200 mg/dL) and HbA1c level > 6.5%. The patients with related anomalies like type 1 diabetes, type 2 diabetes with complications, gestational diabetes mellitus and heart diseases were excluded from the study. The healthy controls who were apparently normal for fasting blood glucose recruited from the Peshawar region. The demographic data including height, weight, gender and family history was obtained from all the participants. Body mass index (BMI) was calculated. Five ml of blood was collected from each subject including patients and healthy controls. Two ml pf the sample was transferred into EDTA vaccutainer and three ml was put in serum separating vaccutainer. The serum was obtained for biochemical analysis and the EDTA whole blood was used for genetic studies. Samples were stored at -20°C till further analyses.

Biochemical analysis and Genotyping of *TCF7L2* gene In all subjects, the biochemical analysis was carried out by measuring the serum fasting glucose and lipid profile (Total cholesterol, Triglyceride, LDL-cholesterol and HDL-cholesterol) by using Clinical Chemistry analyzer. Other biochemical tests including blood HbA1c and creatinine were also determined in all the participants. All the experiments were conducted according to the standard protocols by using commercially available kits. The whole blood of all the subjects was processed for extraction of total genomic DNA by using standard method of phenol-chloroform extraction^24^. The concentration and purity of DNA samples were measured by Nano-drop spectrophotometry (OD at 260/280). Genotype analysis of common SNPs of *TCF7L2* (rs7903146 C>T and rs12255372 G>T) was carried out by using amplification refractory mutation system (AMRS)-PCR techniques17. Four primers were amplified to genotype each SNP, two outers and two inners (rs7903146 C/T; forward inner primer (C allele) 5′-CAATTAGAGAGCTAAGCACTTTTTAGAGAC-3′; reverse inner primer (T allele) 5′-TGCCTCATACGGCAATTAAATTATAGAA-3′; forward outer primer 5′-GTAATGCAGATGTGATGAGATCTCTG-3′; reverse outer primer 5′AGAAAAATACAAAGACATGCAAAAGC-3′ and rs12255372 G/T; forward inner primer (T allele) 5′-CTGCCCAGGAATATCCAGGCAAGAGTT-3′; reverse inner primer (G allele) 5′-GAGGCCTGAGTAATTATCAGAATATGATC-3′; forward outer primer 5′-GGCTGTATGAAGTCATTT-GATGATTGTTT-3′; reverse outer primer 5′-ACGCTTTGAAGGTAGAGAGGACACACT-3′) as described earlier16. For polymerase chain reaction (PCR), total reaction volume was 20 µL containing Master-mix, each inner and outer primers, DNA template and nuclease free water. In thermal cycle, the protocol carries the following cycles; the initial denaturation at 95°C for 5 minutes, then 35 cycles were repeated for denaturation at 94°C for 30 seconds, annealing at 58°C for 30 seconds and cyclic extension at 72°C for 30 seconds and then one cycle of final extension at 72°C for 10 minutes. The amplified products were resolved on 2% agarose gel and the bands were visualized by using ultraviolet (UV) documentation system. The inspection of each band was inferred to determine the genotype (homozygous or heterozygous) patterns.

### Statistical analysis

Data analysis was performed by statistical packages for social sciences (SPSS) version 23. The equation of Hardy Weinberg equilibrium (HWE) was applied to calculate the frequencies of alleles and genotypes for each SNPs of the *TCF7L2* gene. The analysis of continuous quantitative variables was done by independent t test and nominal variables by using Chi-square test (χ2). Two sided chi-square test was used to check the differences in variables and genotype frequencies between T2DM patients and controls. The association of SNPs and risk of T2DM was performed by calculating the odds ratio (OR) and confidence interval (95%) by regression analysis. Statistically, p value was considered significant if it was ≤ 0.05.

## Results

In this study, 176 subjects (118 T2DM cases and 58 controls) were investigated for the genetic association with the disease. The mean demographic (age, BMI) and clinical parameters (fasting glucose, HbA1c, lipid profile, and creatinine) in subjects are presented in Table 1. Most of the variables were significantly different in T2DM cases as compared to controls (p <0.05), while BMI and serum HDL-cholesterol were not different between the groups (p >0.05).

**Table 1:** Comparison of demographic and clinical parameters between T2DM cases and controls of the study

Variables	controls (n=58)	T2DM cases (n=118)	p-value
**Age** (years)	42.81 ± 9.82	57.48 ± 9.09	<0.0001*
**Body Mass Index** (Kg/m^2^)	24.31 ± 3.29	24.66 ± 4.09	>0.567
**Serum fasting** **glucose** (mg/dl)	90.97 ± 6.55	246.53 ± 77.94	<0.0001*
**HbA1c** (%)	5.24 ± 4.66	11.42 ± 2.76	<0.0001*
**Serum T.** **Cholesterol** (mg/dl)	156.31 ± 30.09	177.91 ± 48.02	<0.002*
**Serum Triglyceride** (mg/dl)	145.31 ± 35.38	179.16 ± 66.44	<0.0001*
**Serum LDL-** **cholesterol** (mg/dl)	106.60 ± 25.23	121.23 ± 33.33	<0.004*
**Serum HDL-** **cholesterol** (mg/dl)	33.85 ± 4.49	34.62 ± 8.09	>0.496
**Serum Creatinine** (mg/dl)	0.73 ± 0.16	1.47 ± 2.11	<0.010*

* variables were considered statistically significant when p value < 0.05.

**Fig 1:**
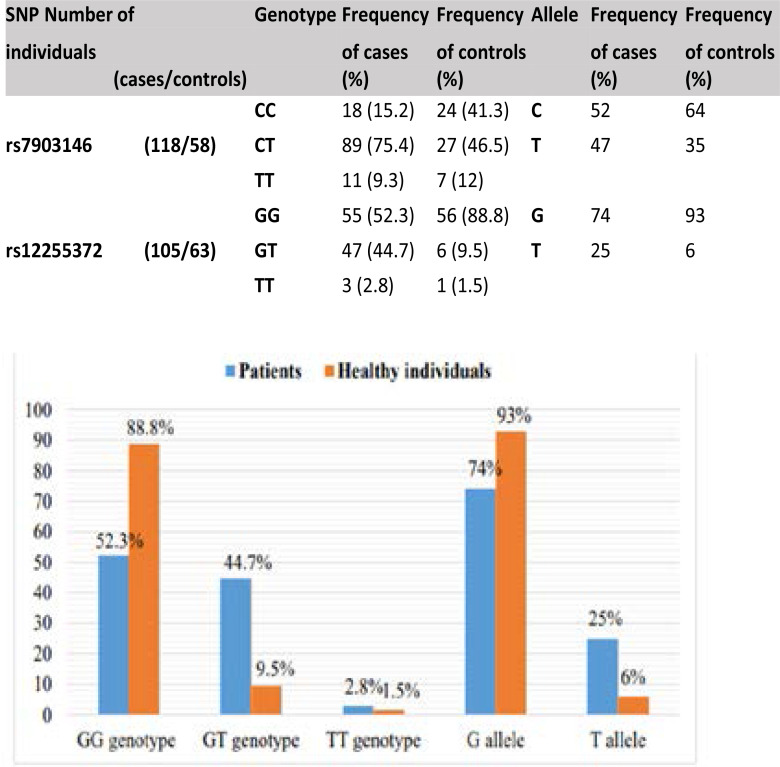
Allele and genotype of SNP rs7903146 of *TCF7L2* in T2DM patients and controls.

**Fig 2:**
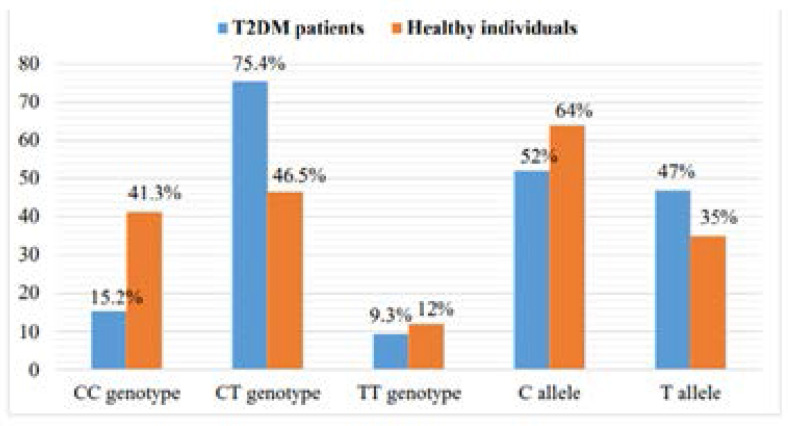
Allele and genotype of SNP rs12255372 of *TCF7L2* in T2DM patients and controls.

The frequencies distribution of the genotypes and the alleles of TCF7L2 SNPs (rs7903146 and rs12255372) were determined by amplifying a sequence specific region in T2DM cases and controls (Table 2). For rs7903146 SNP, the most frequent genotype was CT (75.4%) in patients and was 46.5% in controls). While the allele frequency showed that C allele as most frequent in T2DM cases (52%) and in controls it was 64% (Figure 1). For rs12255372 SNP, the GG genotype distribution was 52.3% in cases and was 88.8% in controls. The G allele frequency was lower (74%)in cases and 93% in controls while, the T allele was found as minor allele (Figure 2) On the other hand, the CT and GT genotypes frequencies were significantly higher in Pakistani cases (75.4% and 44.7% respectively) than in controls (46.5% and 9.5% respectively). However, the frequency of the TT genotypes of both SNPs did not show any significant association with the disease risk (9.3%, 2.8% and 12%, 1.5% respectively).

**Table 2:** Frequencies of genotype and allele distribution of the *TCF7L2* SNPs rs7903146 and rs12255372 in cases and controls

SNP Number of individuals		Genotype	Frequency of cases	Frequency of controls	Allele	Frequency of cases	Frequency of controls
	(cases/controls)		(%)	(%)		(%)	(%)
		**CC**	18 (15.2)	24 (41.3)	**C**	52	64
**rs7903146**	**(118/58)**	**CT**	89 (75.4)	27 (46.5)	**T**	47	35
		**TT**	11 (9.3)	7 (12)			
		**GG**	55 (52.3)	56 (88.8)	**G**	74	93
**rs12255372**	**(105/63)**	**GT**	47 (44.7)	6 (9.5)	**T**	25	6
		**TT**	3 (2.8)	1 (1.5)			

The model analysis was studied to detect the association of *TCF7L2* genetic polymorphism. For SNP rs7903146, association of rs7903146 polymorphism was found with T2DM in the different genetic models. In the co-dominant model, the heterozygous CT contributed 75% occurrence of type 2 diabetes (unadjusted OR = 4.395; 95% CI =2.0810–9.2822; p <0.0001) and in the dominant model (CT/TT) imparted 85% risk (unadjusted OR =3.9216, 95% CI =1.9003–8.0929; p <0.0002) for the disease development. However, no association was observed with TT genotype (unadjusted OR =2.0952; 95% CI =0.6784–6.4707; p >0.1986) and recessive model (unadjusted OR =0.7490, 95% CI =0.2743–2.0454 and p >0.5728) (Table 3). After the adjustment for age, BMI, lipid profile, and other biochemical profiles, the frequency of T allele risk established the strong association of SNP rs7903146 between patients and controls (adjusted OR = 1.6244, 95% CI = 1.0269–2.5694; adjusted p <0.0381).

**Table 3:** Association of rs7903146 SNP of *TCF7L2* gene according to the model of inheritance in patients and controls

Genetic Model/s	Genotype	controls n (%)	cases n (%)	p value	OR (95% CI)	Adjusted P value	Adjusted OR (95% CI)
**Co-dominant**	**CC** (wild-type)	24 (41.3)	18 (15.2)				
	**CT** (Heterozygous)	27 (46.5)	89 (75.4)	<0.0001	4.3951 (2.0810 – 9.2822)	<0.0381	1.6244 (1.0269 – 2.5694)
	**TT** (homozygous)	7 (12)	11 (9.3)		2.0952 (0.6784 – 6.4707)		
**Dominant model**	**CC**	24 (41.3)	18 (15.2)	<0.0002		-	-
	**CT-TT**	34 (58.6)	100 (84.7)		3.9216 (1.9003 – 8.0929)		
**Recessive**	**TT**	7 (12)	11 (9.3)	0.5728	0.7490 (0.2743 – 2.0454)	-	-
	**CC-CT**	51 87.9)	107 (90.6)				
**Over dominant**	**CT**	27 (46.5)	89 (75.4)	<0.0002	3.5236 (1.8126 – 6.8497)	-	-
	**CC-TT**	31 (53.4)	29 (24.5)				

The association of the co-dominant GT genotype (OR =7.9758; 95% CI =3.1544–20.1663; p <0.0001), the dominant model GG vs GT+TT (OR = 7.2727, 95% CI = 3.0344–17.4310 and p <0.0001), and the over-dominant model GT vs GG+TT (OR =7.6983, 95% CI =3.0526–19.4141 and p <0.0001) for the rs12255372 SNP were significantly different between cases and controls (Table 4). However, TT genotype was not associated with T2DM disease (OR =3.0545, 95% CI =0.3082–30.2721 and p =0.3400) and also the recessive model did not link with the phenotype (OR = 1.8235; p = 0.6063) in the studied subjects (Table 4). While, the T allele frequency of rs12255372 SNP was significntly linked between the cases and controls (p =0.0001; OR =4.9793; 95% CI = 2.2806–10.8713).

**Table 4:** Thee genetic model of rs12255372 SNP of *TCF7L2* association in T2DM cases and controls

Genetic Model/s	Genotype	controls n (%)	cases n (%)	p value	OR (95% CI)	Adjusted p value	Adjusted OR (95% CI)
**Co-dominant**	**GG** (wild-type)	56 (88.8)	55 (52.3)				
	**GT** (Heterozygous)	6 (9.5)	47 (44.7)	<0.0001	4.3951 (2.0810 – 9.2822)	< 0.0001	4.9793 (2.2806 – 10.8713)
	**TT** (homozygous)	1 (1.5)	3 (2.8)		2.0952 (0.6784 – 6.4707)		
**Dominant model**	**GG**	56 (88.8)	55 (52.3)	<0.0001		-	-
	**GT-TT**	7 (11.1)	50 (46.7)		3.9216 (1.9003 – 8.0929)		
**Recessive**	**TT**	1 (1.5)	3 (2.8)	0.6063	0.7490 (0.2743 – 2.0454)	-	-
	**GG-GT**	62 (98.4)	102 (97.1)				
**Over dominant**	**GT**	6 (9.5)	47 (44.7)	<0.0001	3.5236 (1.8126 – 6.8497)	-	-
	**GG-TT**	57 (90.4)	58 (55.2)				

## Discussion

T2DM is a complex disease afflicted hundreds of millions in the world and it is increasing rapidly nowadays. This prevalence contributes to the growing urbanization of countries, the sedentary life styles, the environmental changes and the genetic factors. Multiple genes have been studied widely and considered as risk factors for developing T2DM. Among these, *TCF7L2* gene has been elucidated as the strongest risk factor for developing T2DM^25^.

In this study, the association was determined for common SNPs (rs7903146 C/T and rs12255372 G/T) of *TCF7L2* gene with T2DM in Khyber Pakhtunkhwa population. Our results detected the significant association of heterozygous genotypes of both SNPs (CT and GT; p <0.0001) with T2DM susceptibility in T2DM cases. Furthermore, the T allele frequencies for SNPs were also significantly higher in cases than controls (p <0.000). Genetic variations in *TCF7L2* gene has been investigated as risk of T2DM in the diverse populations. In the British ancestry, a study described the nucleotide variations of *TCF7L2* were associated with high risk of disease due to the alterations in pro-insuliconcentrations and impaired function of pancreatic β-cells^26^. The results of present study were in consistent to a previous study in which the T allele frequencies of the SNPs (rs7903146 and rs12255372) were significantly higher in diabetes patients as compared to controls (p <0.00004). Similar results have been reported for rs7903146 SNP in Asian Indian population with type 2 diabetes^27^ and with post-transplant diabetes mellitus^28^. Recently, a meta-analysis study of Indian population described the positive correlation of rs7903146 SNP with gestational diabetes mellitus reported^29^. On the other hand, the difference in genotype distribution of homozygous (TT) and heterozygous genotypes (GT and CT) were associated between T2DM cases and controls^30^, while no association was established for TT genotype in this study. Due to the controversial reports for the involvement of TCF7L2 in T2DM progression but the precise mechanism is still unknown. Though, there are reports suggesting genetic variants of *TCF7L2* may influence the factors for T2DM development by changing the GLP-1 levels indirectly by inducing the gene from transcription factors^31^.

A study from Scandinavian population demonstrated the association of T allele distribution with impaired secretion of insulin due to the proliferation beta cells of pancreas. Various meta-analyses demonstrated the association of common SNPs rs12255372 and rs7903146 as contributing factors for T2DM progression in diverse population like South Asian, Caucasian, East Asian and other ethnicities^16^,^32^,^33^,^34^. On the other hand, various studies from local populations of different countries established the link for disease susceptibility and *TCF7L2* variations^12^,^14^,^35^. Although, the results of present study are in accordance to the previous studies but TT genotypes and recessive genetic models have been found associated in previous study by Wu et al., but other genetic models and the GT genotype did not find any association (p >0.05)^13^. The model analysis results of this study are comparable to the previous study in which co-dominant and over-dominant models were significantly associated with T2DM. Furthermore, there was found association between biochemical parameters and genetic polymorphism except BMI and HDL which are similar to the previous study^16^. In contrary to the current results, several studies did not demonstrated the link between genetic polymorphism of *TCF7L2* and T2DM in different populations and ethnic groups like Chinese population^36^,^37^ and other regions^35^,^38^–^39^. The strength of studying genetic polymorphism of common variants in type 2 diabetes patients of different ethnic groups creates the opportunities to establish the biomarkers for diagnosis and disease management. There are some limitations in this study, sample size, population selection on the basis of ethnicity and advance technologies like DNA sequencing and genome wide sequencing may be helpful to document the genetic variants on large scale.

## Conclusion

It is concluded that the heterozygous genotypes (GT and CT), frequency of T alleles, dominant and over-dominant models of the two common SNPs (rs12255372 and rs7903146) of *TCF7L2* gene are associated with the susceptibility of T2DM in the Northern population of Pakistan. Due to the genetic complexity, there is huge heterogeneity of type 2 diabetes worldwide.

## References

[1] Petersmann A (2019). Definition, Classification and Diagnosis of Diabetes Mellitus. Exp Clin Endocrinol Diabetes.

[2] Aamir AH (2019). Diabetes Prevalence Survey of Pakistan (DPS-PAK): Prevalence of Type 2 Diabetes Mellitus and Prediabetes Using HbA1c: A Population-Based Survey from Pakistan. BMJ Open.

[3] Meo SA (2016). Type 2 diabetes mellitus in Pakistan: Current prevalence and future forecast. J Pak Med Assoc.

[4] Albegali AA (2019). Genetic polymorphism of eNOS (G894T) gene in insulin resistance in type 2 diabetes patients of Pakistani population. International Journal of Diabetes in Developing.

[5] Barnett AH (2006). Type 2 diabetes and cardiovascular risk in the UK south Asian community. Diabetologia.

[6] Sladek R (2007). A genome-wide association study identifies novel risk loci for type 2 diabetes. Nature.

[7] Albegali AA (2019). Association of genetic polymorphism of PC-1 gene (rs1044498 Lys121Gln) with insulin-resistant type 2 diabetes mellitus in Punjabi Population of Pakistan. Mol Genet Genomic Med.

[8] Florez JC (2007). The new type 2 diabetes gene TCF7L2. Curr Opin Clin Nutr Metab Care.

[9] Cauchi S (2007). TCF7L2 is reproducibly associated with type 2 diabetes in various ethnic groups: a global meta-analysis. Journal of Molecular Medicine.

[10] Ding M (2018). Genetic variants of gestational diabetes mellitus: a study of 112 SNPs among 8722 women in two independent populations. Diabetologia.

[11] Zhou Y (2014). TCF7L2 is a master regulator of insulin production and processing. Human Molecular Genetics.

[12] Yao H (2015). Association of TCF7L2 genetic polymorphisms with type 2 diabetes mellitus in the Uygur Population of China. International Journal of Environmental Research and Public Health.

[13] Damcott C (2006). Polymorphisms in the transcription factor 7-like 2 (TCF7L2) gene are associated with type 2 diabetes in the Amish: replication and evidence for a role in both insulin secretion and insulin resistance. Diabetes.

[14] Yang Y (2015). Association of TCF7L2 gene polymorphisms with susceptibility to type 2 diabetes mellitus in a Chinese Hui population. Genetics and molecular research; GMR.

[15] Kalantari S (2019). Single and multi-locus association study of TCF7L2 gene variants with susceptibility to type 2 diabetes mellitus in an Iranian population. Gene.

[16] Siewert S (2015). Association of TCF7L2 Gene Polymorphisms with T2DM in the Population of Juana Koslay, San Luis Province, Argentina. OALib J.

[17] Wu Y (2017). Transcription factor 7-Like 2 rs12255372, rs7903146 and rs290487 polymorphism is associated with the susceptibility to type 2 diabetes mellitus in a Chinese population. Int J Clin Exp Pathol.

[18] Ganmore I (2019). TCF7L2 polymorphisms are associated with amygdalar volume in elderly individuals with Type 2 Diabetes. Sci Rep.

[19] Wunsch C (2019). Lack of association between TCF7L2 gene variants and type 2 diabetes mellitus in a Brazilian sample of patients with the risk for cardiovascular disease. Endocr Regul.

[20] Shahid S (2019). Lack of Genetic Association between SNP Rs7903146 (Ivs3c>T) of Transcription Factor 7 Like 2 (Tcf7l2) Gene in Type 2 Diabetes Mellitus in Pakistani Population. PJMHS.

[21] Dalhat MH (2017). Association of rs7903146 TCF7L2 (C/T) Gene Polymorphism and Type 2 Diabetes Mellitus in Pakistani Population. Journal of Applied Life Sciences International.

[22] Shahzadi S (2019). Genome-wide implicated risk variants of TCF7L2 gene contribute to type 2 diabetes susceptibility by modulating serum lipids in Pakistani population. International Journal of Diabetes in Developing Countries.

[23] Rees SD (2008). Common variants of the TCF7L2gene are associated with increased risk of type 2 diabetes mellitus in a UK-resident South Asian population. BMC Med Genet.

[24] Green MR (2017). Isolation of high-molecular-weight DNA using organic solvents. Cold Spring Harbor Protocols.

[25] Van De Wetering M (2002). The β- catenin/TCF-4 complex imposes a crypt progenitor phenotype on colorectal cancer cells. Cell.

[26] Loos RJ (2007). TCF7L2 polymorphisms modulate proinsulin levels and β-cell function in a British Europid population. Diabetes.

[27] Khan IA (2015). Type 2 Diabetes Mellitus and the Association of Candidate Genes in Asian Indian Population from Hyderabad, India. J Clin Diagn Res.

[28] Khan IA (2015). Validation of the association of TCF7L2 and SLC30A8 gene polymorphisms with post-transplant diabetes mellitus in Asian Indian population. Intractable Rare Dis Res.

[29] Khan IA (2015). Genetic confirmation of T2DM meta-analysis variants studied in gestational diabetes mellitus in an Indian population. Diabetes Metab Syndr.

[30] van Vliet-Ostaptchouk J (2007). Association of variants of transcription factor 7-like 2 (TCF7L2) with susceptibility to type 2 diabetes in the Dutch Breda cohort. Diabetologia.

[31] Yi F (2005). TCF-4 mediates cell type-specific regulation of proglucagon gene expression by β-catenin and glycogen synthase kinase-3β. Journal of Biological Chemistry.

[32] Guan Y (2016). Correlation of the TCF7L2 (rs7903146) polymorphism with an enhanced risk of type 2 diabetes mellitus: a meta-analysis. Genet Mol Res.

[33] Ding W (2018). Meta-analysis of association between TCF7L2 polymorphism rs7903146 and type 2 diabetes mellitus. BMC Med Genet.

[34] Peng S (2012). TCF7L2 gene polymorphisms and type 2 diabetes risk: a comprehensive and updated meta-analysis involving 121 174 subjects. Mutagenesis.

[35] Saadi H (2008). Association of TCF7L2 polymorphism with diabetes mellitus, metabolic syndrome, and markers of beta cell function and insulin resistance in a population-based sample of Emirati subjects. Diabetes Research and Clinical.

[36] Ren Q (2008). Exon sequencing and association analysis of polymorphisms in TCF7L2 with type 2 diabetes in a Chinese population. Diabetologia.

[37] Barros C (2014). Association of the rs7903146 and rs12255372 polymorphisms in the TCF7L2 gene with type 2 diabetes in a population from northeastern Brazil. Genet Mol Res.

[38] Pourahmadi M (2015). Non-association between rs7903146 and rs12255372 polymorphisms in transcription factor 7-Like 2 gene and type 2 diabetes mellitus in Jahrom city, Iran. Diabetes & Metabolism Journal.

[39] Guo T (2007). TCF7L2 is not a major susceptibility gene for type 2 diabetes in Pima Indians: an analysis of 3501 individuals. Diabetes.

